# Supplementary material for: Evolutionary origins and functional diversification of Auxin Response Factors
Source: Nat Commun. 2024 Dec 30;15:10909. doi: 10.1038/s41467-024-55278-8 (PMC11685440; doi:10.1038/s41467-024-55278-8)
Supplement: Supplementary file 3 — Description of Additional Supplementary Files [file 41467_2024_55278_MOESM3_ESM.pdf]

Description of additional Supplementary Files

Supplementary Data 1. Phmmer output hit proteins with different AD and DD-AD sequence queries.

Supplementary Data 2. β-galactosidase assays data for Figure 2g and Figure 5e.

Supplementary Data 3. Genomic databases used in the study.

Supplementary Data 4. Plasmids used in the study.

Supplementary Data 5. Oligonucleotide sequences used in the study.
